# TGF-β phospho antibody array identifies altered SMAD2, PI3K/AKT/SMAD, and RAC signaling contribute to the pathogenesis of myxomatous mitral valve disease

**DOI:** 10.3389/fvets.2023.1202001

**Published:** 2023-10-16

**Authors:** Andrew J. McNair, Greg R. Markby, Qiyu Tang, Vicky E. MacRae, Brendan M. Corcoran

**Affiliations:** ^1^The Roslin Institute, The University of Edinburgh, Easterbush Veterinary Centre, Roslin, United Kingdom; ^2^Royal (Dick) School of Veterinary Studies, The University of Edinburgh, Easterbush Veterinary Centre, Roslin, United Kingdom

**Keywords:** myxomatous mitral valve disease, transforming growth factor-beta, canine, SMAD2, PI3K/AKT/mTOR

## Abstract

**Background:**

TGFβ signaling appears to contribute to the pathogenesis of myxomatous mitral valve disease (MMVD) in both dogs and humans. However, little is known about the extent of the downstream signaling changes that will then affect cell phenotype and function in both species.

**Objective:**

Identify changes in downstream signals in the TGFβ pathway in canine MMVD and examine the effects of antagonism of one significant signal (SMAD2 was selected).

**Materials and methods:**

Canine cultures of normal quiescent valve interstitial cells (qVICs) and disease-derived activated myofibroblasts (aVICs) (*n* = 6) were examined for TGFβ signaling protein expression using a commercial antibody array. Significant changes were confirmed, and additional proteins of interest downstream in the TGFβ signaling pathway and markers of cell phenotype were examined (PRAS40, S6K, elF4E IRS-1, αSMA, and VIM), using protein immunoblotting. RT-PCR examined expression of gene markers of VIC activation (*ACTA2, TAGLN*, and *MYH10*; encoding the proteins αSMA, SM22, and Smemb, respectively). Attenuation of pSMAD2 in aVICs was examined using a combination of RNA interference technology (siRNA) and the SMAD7 (antagonizes SMAD2) agonist asiaticoside.

**Results:**

The antibody array identified significant changes (*P* < 0.05) in 19 proteins, of which six were phosphorylated (p). There was increased expression of pSMAD2 and pRAC1 and decreased expression of pmTOR, pERK1/2, and pAKT1. Expression of pPRAS40 and pIRS-1 was increased, as was the mTOR downstream transcription factor pS6K, with increased expression of peIF4E in aVICs, indicating negative feedback control of the PI3K/AKT/mTOR pathway. SMAD2 antagonism by siRNA and the SMAD7 agonist asiaticoside decreased detection of pSMAD by at least 50%, significantly decreased expression of the aVIC gene markers *ACTA2, TAGLN*, and *MYH10*, and pαSMA, pAKT2, and pERK1, but had no effect on pS6K, pERK2, or pVIM expression in aVICs. SMAD2 antagonism transitioned diseased aVICs to normal qVICs, while maintaining a mesenchymal phenotype (VIM+) while concurrently affecting non-canonical TGFβ signaling.

**Conclusion:**

MMVD is associated with changes in both the canonical and non-canonical TGFβ signaling pathway. Antagonism of SMAD2 transitions diseased-activated myofibroblasts back to a normal phenotype, providing data that will inform studies on developing novel therapeutics to treat MMVD in dogs and humans.

## 1. Introduction

Myxomatous mitral valve disease (MMVD) is the single most important acquired cardiovascular disease in dogs and shares close similarities with analogous human conditions (1, 2). It is a major cause of morbidity and mortality in affected dogs and causes significant financial and emotional stress for owners. The disease is so prevalent that most elderly dogs show some evidence of the disease, but it is predominantly seen in small breed dogs, with a greater preponderance in certain predisposed breeds, the Cavalier King Charles Spaniel being the best example (2, 3). The development and progression of MMVD in terms of the pathological changes in the mitral valve, the hemodynamic consequences including cardiac remodeling, the clinical progression, and the therapeutic options when congestive heart failure develops are well-described. In addition, much is known about valve changes at the ultrastructural and cell level. Briefly, the disease involves the gradual development of myxomatous degeneration over several years with disorganization of collagen bundles, reduction in collagen content, and excess production of glycosaminoglycans (4–7). This results in distorted valve architecture and geometry with poor coaptation of leaflets, allowing mitral valve regurgitation and development of a characteristic murmur. The main cell changes include the transition of quiescent valve interstitial cells (qVICs) to an activated myofibroblast phenotype (aVICs), as evidenced by increased expression of αSMA, SM22, and Smemb, and valve endothelial cell damage and loss (8–11). The appearance of αSMA positive VICs is a cardinal feature of the disease, and this activated myofibroblast cell type is believed to control the aberrant extra cellular matrix (ECM) remodeling characteristic of the disease.

At the molecular level, transcriptomic profiling in both valve tissue and cultured aVICs has identified a range of gene changes, most notably in the TGFβ signaling pathway and ECM genes (12–15). In addition, there is up-regulation of the *5HT2B* receptor gene, but since serotonin (5HT) itself has not been shown to transition qVICs, this likely reflects the activity of TGFβ (16). 5HT itself can induce VIC proliferation and ECM production through the activation of ERK1/2, a downstream component of the TGFβ signaling pathway (17, 18). Strong evidence suggests the TGFβ pathway is one of the most important in the pathogenesis of MMVD and in the control of the VIC phenotype. For example, TGFβ antagonism by SB431542 transitions canine aVICs back to a more normal qVIC phenotype (16). Examining further changes in the downstream parts of the TGFB pathway will give insight into the molecular mechanisms controlling disease and allow the identification of potential novel therapeutic targets to control disease development and progression.

Evidence for TGFβ involvement in MMVD can also be seen in the analogous human disease (Barlow's Disease) and various animal models (19–22). This involves both canonical (SMAD2/3) and non-canonical downstream signaling pathways, as demonstrated by several knockout mouse models of MMVD, including the Fbn-1 (fibrillin-1; Marfan syndrome), FLN-A (filamin-A X-linked), and Fstl1 (follistatin) mouse models (23–25). All these models show myxomatous degeneration, changes in TGFβ signaling and downstream signals, and expression of the aVIC marker αSMA. Most of these models have clarified the contribution of canonical SMAD2/3 signaling to MMVD. However, there is evidence that non-canonical signaling including the mitogen-activated protein kinases TAK1, JNK, and ERK1/2 and the PI3K kinase can also contribute to MMVD pathogenesis, and through molecular cross-talk affect SMAD2/3 signaling (24, 26). Furthermore, transcriptomic profiling has found changes in *ERK1/2* gene expression in canine valves, mouse models, and human aVICs, and the effects on and interaction between canonical and non-canonical pathways can be clearly seen.

However, the interplay between these pathways is only partially understood and to what extent one alone might dominate to affect aVIC phenotype, and by extension ECM remodeling, is unknown. In this study, the aim was to examine expression of key molecules in the downstream TGFβ signaling pathway in canine aVICs using an antibody bio-array approach, measuring the expression of both total and phosphorylated proteins. This would be followed by confirming such changes using protein immunoblotting and then selecting one target of interest for antagonism, examining the effects on aVIC phenotype.

## 2. Materials and methods

### 2.1. Valve samples and cell culture

Mitral valves were collected with full informed owner consent from canine patients presented for euthanasia at the Hospital for Small Animals, R(D)SVS, the University of Edinburgh and with full ethical approval for valve tissue collection from the Veterinary Ethics in Research Committee of the R(D)SVS (VERC# 96/21). Valves were processed for the culture of valve interstitial cells (VICs) using our previously reported protocols, using a validated low serum (2% FBS) culture media method and not used beyond passage eight (16). Cells were grown on 75 cm^3^ plates and were harvested when they reached ~80% confluence.

VICs isolated from six normal (quiescent phenotype) and diseased (activated phenotype) dog mitral valves were grown in T75 flasks before being split into 100 mm^2^ cell culture dishes and grown to confluence. Despite confirmed disease status (canine valves are graded normal 0 to severe 4), we have previously identified heterogeneity in valve interstitial cell phenotype. To confirm the consistent phenotype for both groups we examined cells for the expression of *ACTA2* (αSMA), *TAGLN* (SM22), and *MYH10* (SMemb) (standard markers of VIC phenotype) from our total archive of 50 until the requisite six per group were identified. The details of the 12 dogs are shown in Table 1.

**Table 1:** Summary of cells used in array analysis.

**Breed**	**Cell ID**	**Grade of disease**	**Age**	**Gender**	**Passage number**
Rottweiler	RotG0	0	6	Male	2
Beagle	D10	0	2	Male	2
German Shepherd	GSG0	0	5	Female	2
Beagle	BG0	0	3	Female	3
Pitbull Cross	PRGo0	0	3	Male	2
Beagle	d09	0	3	Female	4
Staffordshire Bull Terrier	STG2	2	5	Female	2
Labrador	D2	2	6	Male	3
Lurcher	LURG2	2	5	Male	2
American Pitbull Cross	APBG2	2	7	Male	2
Beagle	BG2	2	5	Female	2
Cavalier King Charles Spaniel	CKCSG4	4	15	Male	3

### 2.2. Full moon biosystems TGFB array

To examine a large set of phosphorylated proteins in the TGFβ pathway, a MAP kinase signaling antibody array was used (Full Moon Biosystems, Sunnyvale, CA, USA). Full details can be found at this site https://www.fullmoonbio.com/product/tgfb-phospho-antibody-array/). The array includes 63 antibodies with six replicates per antibody; details of the antibodies are show in Table 2. All procedures with the array followed the manufacturer's instructions (Supplementary material 1) and example of the microarray slide shown in Supplementary Figure 1.

**Table 2:** Full Moon TGFβ MAP kinase signaling antibody array.

**ID**	**Antibody**	**Reactivity**	**Swiss Prot**
55	AKT1	HMR	P31749
39	AKT2	HMR	P31751
2	Akt3	H	Q9Y243
59	A-RAF	HMR	P10398
25	ASK1	H	Q99683
11	ASK2	H,M	O95382
44	ASK3	H	Q6ZN16
16	BRAF	H	P15056
60	c-RAF	HMR	P04049
61	CREB	HMR	P16220
1	Elk1	H,M,R	P19419
20	ERK1	H	P27361
33	ERK2	H	P28482
22	ERK3	H	P16659
21	ERK4	H	P31152
9	ERK7	H	Q8TD08
10	GSK3 alpha	H	P49840
15	GSK3 beta	H,M,R	P49841
52	HGK	H	O95819
63	HSP27	H	P04792
29	JNK1	H	P45983
53	JNK2	H	P45984
41	JNK3	H	P53779
46	KHS1	H	Q9Y4K4
7	KHS2	H,M,R	Q8IVH8
23	LAMTOR3	H	Q9UHA4
3	MAP2K4	H	P45985
36	MEK1	H	Q02750
40	MEK2	H	P36507
24	MEK3	H	P46734
57	TAK1	H	O43318
56	MEK5	H	Q13163
48	MEK6	H	P52564
49	MEKK1	H	Q13233
51	MEKK3	H	Q99759
45	MEKK4	HM	Q9Y6R4
8	MINK	H,M	Q8N4C8
47	MK2	H	P49137
27	MK3	HM	Q16644
43	MLK	H	O43283
12	MLK1	H,M	P80192
13	MLK2	H,M	Q02779
50	MLK3	H	Q16584
6	MLK4	H	Q5TCX8
35	MSK1	H	O75582
62	mTOR	H	P42345
14	NF-kB p65	H,M	Q04206
28	p38A	H	Q16539
18	p38B	H	Q15759
42	p38D	H	O15264
19	p38G	H	P53778
5	p44/42MAPK	H,M,R	P27361/P28482
4	p53	H,M,R	P04637
54	p70S6K	H	P23443
32	p70S6K2	H	Q9UBS0
26	PRAK	H	Q8IW41
38	RSK1	H	Q15418
31	RSK2	H	P51812
37	RSK3	H	Q15349
30	RSK4	HR	Q9UK32
17	RSKL1	H	Q96S38
34	STAT1	H	P42224
58	TAB1	HR	Q15750

### 2.3. Protein immuno-blotting (western blotting)

To confirm the array data findings, and to examine other proteins of interest, samples were examined for total and phosphorylated protein expression using protein immuno-blotting (Western Blotting, WB) using a standard protocol. Details of the methodology are shown in Supplementary material 2. All antibodies used for WB are shown in Supplementary Table 2. We used the total expression of each individual phospho experiment as the loading control, as per previous work. In the experiments where we had no totals, β-actin was used as a loading control. In terms of exposure times, we used an automated system (GeneGnome, Syngene, Cambridge, UK) that set the optimal exposure timepoints; it was not something that could be edited by us.

### 2.4. Gene expression analysis

#### 2.4.1. RNA extraction

To extract RNA from VICs, lysis was performed using QIAzol lysis reagent and extracted with the miRNEASY extraction kit according to manufacturer's instructions. Initially, 700 μl QIAzol was added to the cells, and they were scraped as previously described. For RNA extraction, chloroform was added and tubes shaken vigorously for separation of RNA from DNA, proteins, and lipids. The RNA was then precipitated with 100% RNA-free ethanol. The sample was transferred onto a spin column to undergo spin column-based nucleic acid purification. Samples were analyzed by a NanoDrop, ND-1000 spectrophotometer (Thermo Scientific, UK) to determine RNA concentrations.

#### 2.4.2. Reverse transcription

Extracted RNA samples were reverse transcribed using TaqMan^®^ reverse transcription reagents (Applied Biosystems, CA, USA) as per manufacturers' instructions. Approximately 1 μg RNA was transcribed per sample in a cocktail of Reverse Transcriptase buffer, 25 mM magnesium chloride, deoxynucleotide triphosphates, random hexamers, RNase inhibitors, and Multiscribe. Reverse transcription was performed using the Veriti^®^ Thermal Cycler (Life Technologies, Paisley, UK) under the following cycling conditions: 10 min at 25°C to maximize primer RNA template binding, 30 min at 48°C for reverse transcription, and 5 min at 95°C to deactivate reverse transcription.

#### 2.4.3. Quantitative real-time polymerase chain reaction

Quantitative real-time polymerase chain reaction was used to assess mRNA expression. Taqman^®^ PCR master mix and fluorescently tagged Taqman^®^ primers (Supplementary Table 1) (Primer design, Southampton, UK) were used. Fluorescence was measured using a real-time PCR System (Life Technologies, Paisley, UK). The cycle conditions are 50°C for 2 min, 95°C for 10 min and 40 cycles of 95°C for 15 s, and 60°C for 1 min. Primer sequences used can be found in our previous paper [(16), p. 1841].

### 2.5. SMAD 2 antagonism

#### 2.5.1. SMAD2 siRNA

siRNA duplex sequences targeted to SMAD2 (TriFECTa^®^RNAi Kit Integrated DNA Technologies; Tyne & Wear, UK) were used to knockdown SMAD2 gene and protein expression. A scrambled siRNA was used as control. siRNA transfection was carried out using lipofectamine 3000 transfection reagent (Invitrogen, Paisley, UK). Lipofectamine 3000 was mixed with the siRNA and incubated for 15 min at room temperature, allowing complexes to form. VICs were then transfected with a final concentration of 5 nM siRNA. Transfection of VICs with siRNA targeting SMAD2 resulted in at least a 50% reduction in SMAD2 protein expression. The siRNA sequence was 5′ TTTTGTAATACGACTCACTATAGGGCGGCCGGGAATTCGTCGACTGGATCCGGTACCGAGGAGATCTGCCGCCGCGATCGCC 3′.

#### 2.5.2. Asiaticoside

The SMAD7 agonist asiaticoside (AST; Cambridge Bioscience Limited, Cambridge, UK) was used to increase SMAD7 expression, leading to an inhibition of pSMAD2. AST was prepared in DMSO, and the vehicle was used as control. AST was added to the VICs at a concentration of 500 mg/l for an incubation period of 3 days (27). Incubation with AST reduced SMAD2 protein phosphorylation expression by at least 50%. The concentration and incubation period for AST and the siRNA were determined using both preliminary dose response and time course experiments as well as using published methodology using the same reagents or siRNA (28, 29).

## 3. Results

### 3.1. Full moon biosystem TGF-β phospho antibody array

Considering the dataset as a whole, significant (*P* < 0.05) changes in total protein in 21 antibodies targeted against 19 different proteins were found (Table 3). This included six antibodies targeted against phosphorylated proteins where a constituent decrease in phosphorylated protein was shown. Overall changes in various different downstream TGFβ pathway molecules were detected, including members of the PI3K/AKT/mTOR, JNK, ERK, and SMAD2 pathways.

**Table 3:** Significantly (*P* < 0.05) altered total protein changes in the TGFβ pathway.

**Total protein**	**Average normal signal intensity**	**Normal SEM**	**Average disease signal intensity**	**Diseased SEM**	**Fold change**	***P*-value**
Myc	2.49	0.06	1.72	0.09	−1.45	0.2
Cofilin	1.13	0.02	0.85	0.035	−1.33	0.03
mTOR (Phospho Ser3)	8.87	0.39	4.79	0.51	−1.85	0.04
SAPK/JNK	1.42	0.047	1.04	0.01	−1.36	0.01
p38MAPK	0.28	0.01	0.49	0.02	1.73	0.02
PKC theta	0.91	0.19	0.74	0.017	−1.22	0.03
Rac1/cdc42	2.37	0.50	1.6	0.87	−142	0.02
SP1	0.41	0.014	0/7	0.04	1.9	0.009
ERK8	0.39	0.01	0.7	0.03	1.83	0.009
RhoA	19.23	1.01	9.29	0.79	−2.06	0.01
AKT1	0.29	0.01	0.73	0.07	2.5	0.04
PAK4/5/6	1.59	0.02	1.168	0.03	−1.37	0.004
PKC alpha	0.18	0.017	0.5	0.036	2.79	0.01
PKC zeta	0.19	0.027	0.52	0.02	2.71	0.005
Smad2 (Ab255)	15.0	0.11	10.06	0.56	−1.49	0.008
Smad2 (Ab245)	0.13	0.02	0.56	0.05	4.27	0.02
mTOR (Ab2446)	0.03	0.02	0.3	0.03	9.17	0.03
TGFβ3	0.39	0.033	0.78	0.047	1.98	0.03
JNNK (MKK4)	0.89	0.025	0.4	0.04	4.59	0.03
S6K-alpha 6	0.136	0.02	0.397	0.03	2.91	0.04
RASE	1.51	0.042	1.21	0.025	−1.25	0.04

Significant changes were also detected in six phosphorylation states with two increased and four decreased in the diseased cells. The phosphorylation changes detected also match with many of the pathways detected to change at the total protein level in the diseased cells, indicating that these pathways may be of some importance in pathogenesis.

Since the *P* < 0.05 cut off value is somewhat arbitrary in an experiment where hundreds of comparisons are being performed simultaneously, and to reduce missing proteins of interest by chance, by expanding the inclusion criteria to *P* < 0.1 in the total protein dataset, changes in an additional 22 antibodies against 17 different proteins were identified (Supplementary Table 1). Examining these proteins more closely, there tended to be a larger variance in the diseased group than the normal (as exampled by the SEMs), with typically 1–3 of the samples in this group showing a large difference to other members of the group.

Conversely, when expanding the same inclusion criteria in the phosphorylated protein dataset, only two more proteins (SP1 and PI3-kinase p85-alpha both downregulated in disease) were identified (Supplementary Table 2). However, throughout the dataset there were several proteins that showed a large fold change (all increasing in disease) but did not reach significance set at *P* < 0.05. Again, this appears to be due to a larger variance in the diseased group. In general, there appeared to be a greater variation within groups in the phosphorylated dataset than in the total protein dataset.

### 3.2. Protein immuno-blotting

**Figure 1:**
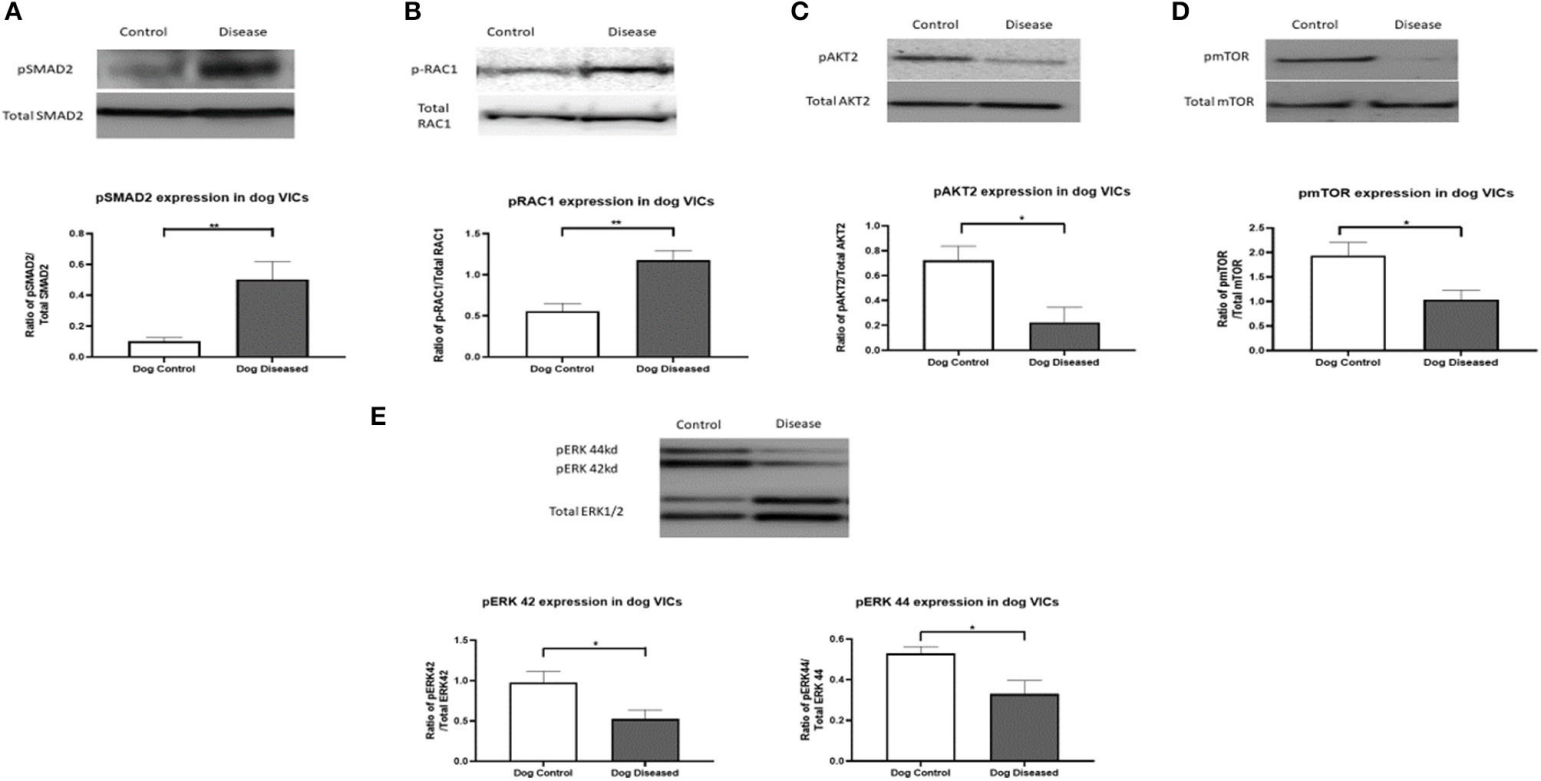
Protein expression of pRAC1 (28 kDa), pSMAD2 (52 kDa), pAKT2 (60 kDa), pmTOR (289 kDa), and pERK1/2 (42/44 kDa) in canine valve interstitial cells. Western blot analysis was used to measure protein expression in qVICs and aVICs. In aVICs pSMAD **(A)**, pRAC1 **(B)** was increased, whilst pAKT **(C)**, pmTOR **(D)**, and pERK1/2 **(E)** were decreased compared to the qVICs. Data expressed as mean + SEM and analysis carried out by *t*-test. *P < 0.05 (*n* = 6), **p < 0.01.

To confirm the antibody array data for the significantly altered phosphorylated proteins targeted, protein immunoblotting was undertaken for pERK1/2, pRAC1, pSMAD2, pAKT2, and pmTOR. For a loading control, the total expression of each of the targeted proteins were used and then a phosphorylation/total expression ratio was calculated for analysis. Results for all five proteins matched the results observed in the array. Phosphorylation of SMAD2 and RAC1 was increased in canine aVICs and phosphorylation of ERK1/2, AKT2, and mTOR protein was more decreased in the aVICs compared to the qVICs (Figure 1).

**Figure 2:**
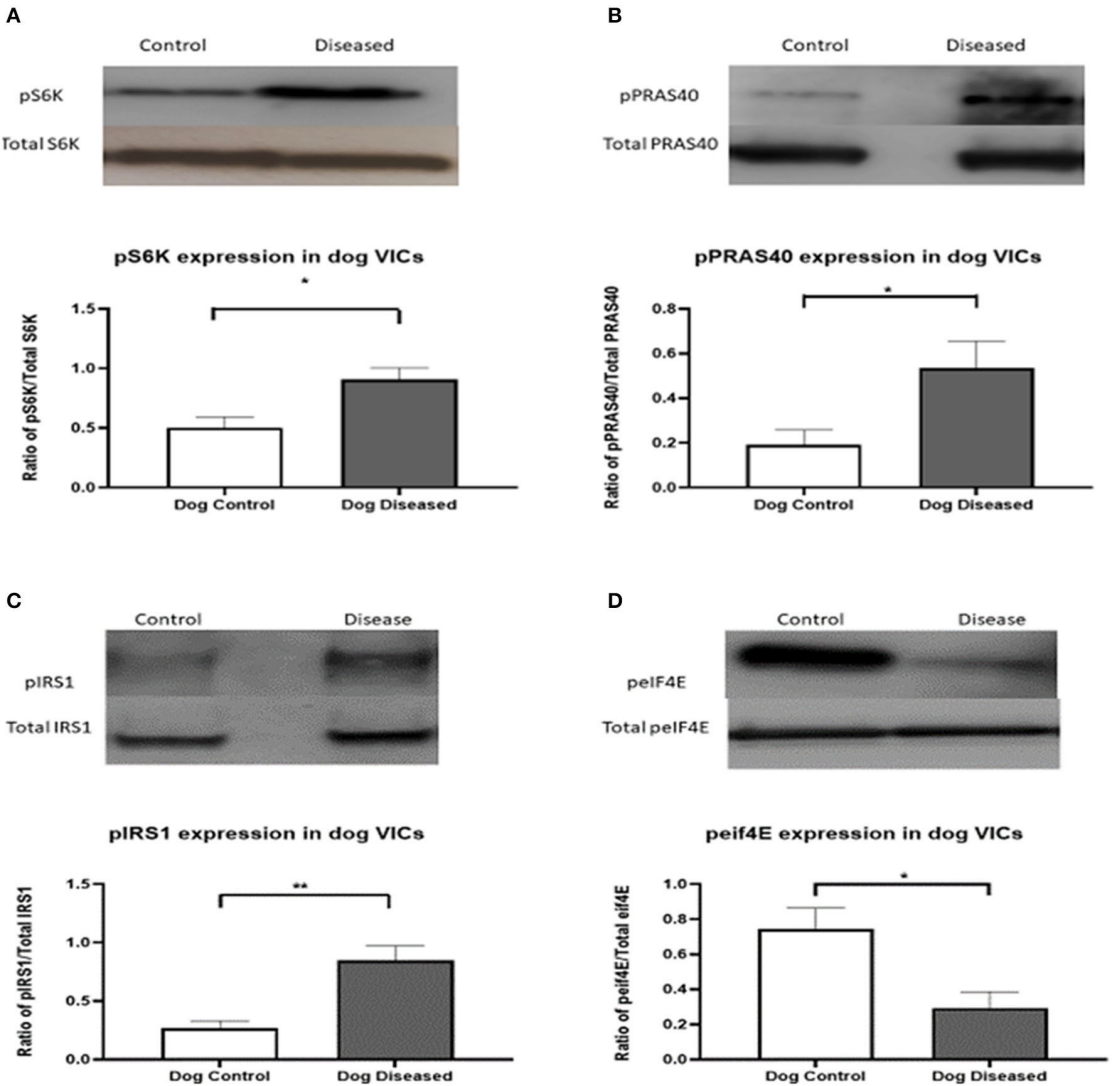
Protein expression of pS6K (70 kDa), pPRAS40 (40 kDa), pIRS-1, and peIF4E (25 kDA) in canine valve interstitial cells. Western blot analysis was used to measure protein expression in qVICs and aVICs. In aVICs pS6K **(A)**, pPRAS40 **(B)**, and pIRS-1 **(C)** was increased, whilst peIF4E **(D)** was decreased compared to the qVICs. Data expressed as mean + SEM and analysis carried out by *t*-test. *P < 0.05, **P < 0.01 (*n* = 6).

For further analysis, and since a novel finding that the PI3K/Akt/mTOR pathway appeared to be affected in aVICs, we decided to analyze this pathway in more detail using WB examining the expression of the downstream transcription factors S6K and elF4E, and the negative feedback controllers of this pathway, IRS-1 and PRAS40. In aVICs detection of pS6K, pPRAS40, and pIRS-1 was significantly increased compared to qVICs while peIF4E was significantly decreased (Figure 2).

### 3.3. Effects of SMAD2 inhibition using siRNA and the SMAD7 agonist asiaticoside

**Figure 3:**
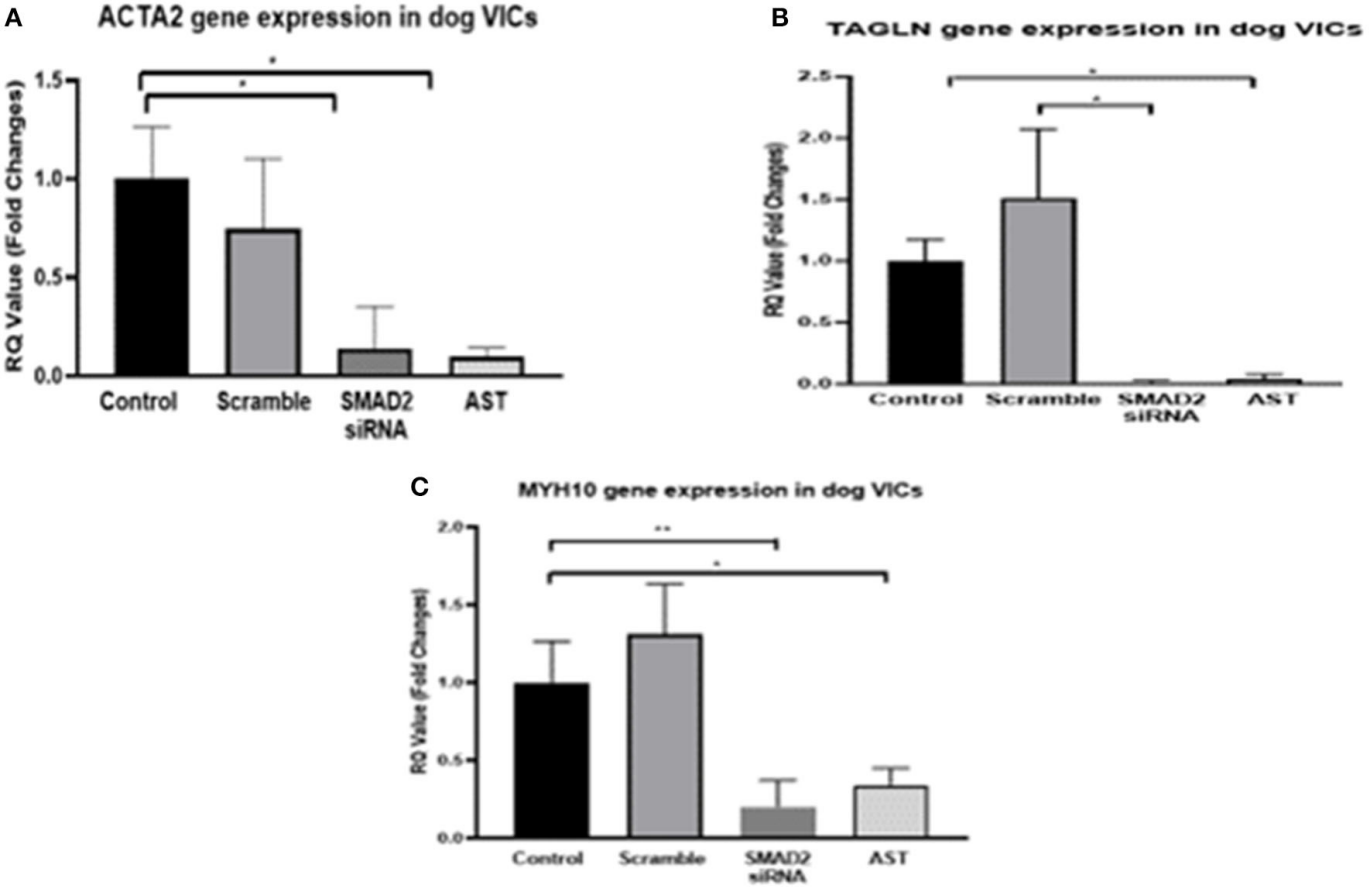
Gene expression of MMVD markers in aVICs. Cells were quiesced for 24 h, 10 nM siRNA or 500 mg/L AST was added to the cells and incubated for 3 days, and then mRNA was extracted for q-PCR. Pharmacological inhibition of SMAD2 resulted in a decreased expression of *ACTA2* **(A)**, *TAGLN* **(B)**, and *MYH10* **(C)** in the aVICs. Data expressed as mean + SEM and analysis carried out by two-way ANOVA with a Tukey *post-hoc* test. *P < 0.05, **P < 0.01 (*n* = 6).

Since our secondary aim was to identify methods to modify disease cell phenotype and we had previously shown TGFβRII receptor antagonism will switch aVICs to a more normal qVIC phenotype, we decided to examine the effects of SMAD2 inhibition on cell phenotype, protein, and gene expression using a combination of SMAD2 siRNA and the SMAD7 agonist asiaticoside (16). In the cells treated with SMAD2 siRNA and AST, there was a significant reduction in the expression of *ACTA2, TAGLN*, and *MHY10* compared to both the diseased control and the scramble siRNA control, indicating revision to a more normal quiescent phenotype (qVIC) (Figure 3).

**Figure 4:**
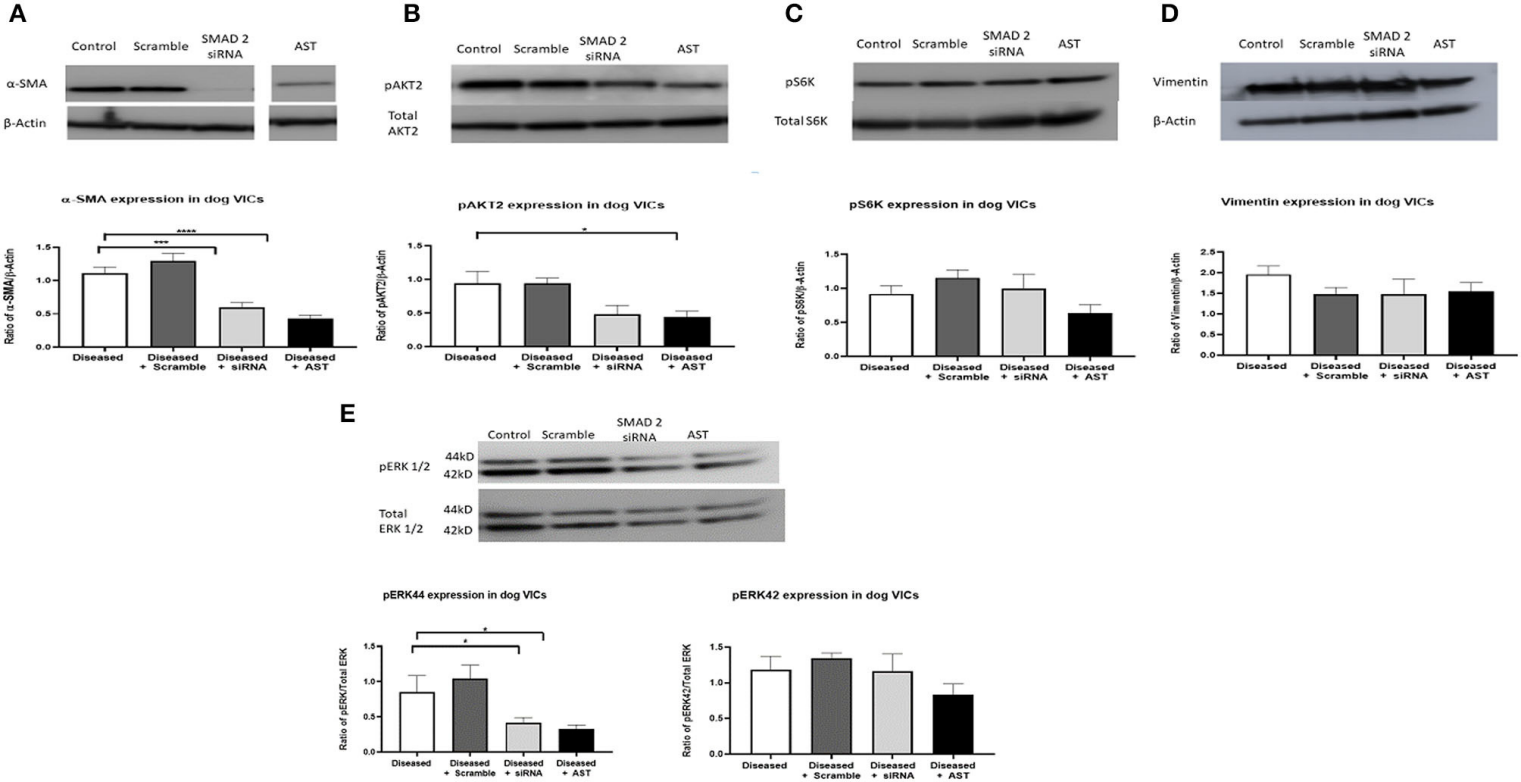
Protein expression of MMVD markers and downstream TGF-β molecules in activated valve interstitial cells. Cells were quiesced for 24 h, and then 10 nM siRNA or 500 mg/L AST was added to the cells and incubated for 3 days. Protein was extracted for Western blotting. Pharmacological inhibition of SMAD2 resulted in a decreased expression of pSMAD2 and αSMA **(A)**. pAKT2 protein expression was decreased in cells treated with AST **(B)** while expression of pS6K **(C)** and Vimentin **(D)** remained unchanged. The 44 kD band of ERK was decreased, however those changes were not observed for the 42 kD band **(E)**. Data expressed as mean + SEM and analysis carried out by two-way ANOVA with a Tukey *post-hoc* test. *P < 0.05, ***p < 0.001, and ****p < 0.0001. α-SMA (42 kDa), β-actin (42 kDa).

SMAD2 siRNA and AST decreased the expression of SMAD2 and the corresponding phosphorylation of SMAD2 compared to the diseased controls and the scramble siRNA groups. There was also a decrease in α-SMA protein expression in both SMAD2 siRNA and AST groups and a decrease in phosphorylation of AKT2, however this occurred in the AST group only. Finally, pS6K and vimentin (a stable marker of mesenchymal cell phenotype) protein expression remained unchanged comparing all groups. For ERK1/2, the 44 kD band (ERK1) was decreased in the siRNA and AST cells, however the 42 kD band (ERK2) remained unchanged (Figure 4).

## 4. Discussion

This study aimed to investigate the signaling downstream of TGFβ1 in diseased VICs compared to normal quiescent VICs in canine MMVD. By interrogating diseased valve interstitial cells using a commercially available protein antibody array, we were able to identify changes in SMAD2 expression and to show antagonism would revert cells back to a normal phenotype. We have also shown significant differences in both total and phosphorylated proteins for several of the non-canonical components of the TGFβ signaling pathway. These results can be used to inform future studies and interrogation of these pathways to confirm their role in disease pathogenesis and examine for potential novel therapeutics. One dog included had advanced disease but had a similar response to that from dogs with milder disease. This would suggest the changes are consistent irrespective of disease status, but further studies would be required to confirm this.

One pathway that consistently changed in all forms of analysis (both increased and decreased) was non-canonical PI3K/AKT/mTOR. This pathway has been associated with various context-specific effects on cells including transformation into a more mesenchymal phenotype, inducing and inhibiting apoptosis, and matrix protein expression (30–33). In the data found here there appears to be a downregulation in the phosphorylation of components of this pathway. It may be the case that a general decrease in phosphorylation is indicative of the change in phenotype between diseased and healthy cells, however what this means is difficult to conclude from the data. One explanation for this finding is negative feedback in diseased cells being constitutively active for prolonged periods. By further examining changes in the PI3K/AKT/mTOR pathway looking at the downstream transcription factors S6K and eIF4E, and PRAS40 (inhibits mTOR and IRS-1), we could identify their role in the negative feedback of the PI3K pathway, an effect commonly seen in various cancers and cancer models (27, 34–36). Reduced elF4E, in combination with increased pPRAS40, will repress apoptosis, and by PRAS40 limiting mTOR activation will also reduce autophagy (37, 38). This negative feedback loop involves S6K phosphorylating insulin receptor substrate 1 (IRS-1). IRS-1 then inhibits PI3K, leading to a decreased expression of the downstream molecules. However, considering what is found in cancer models, this negative feedback is not activated in early-stage disease, permitting eIF4E and S6K to act on the downstream regulators of mesenchymal cell transition, apoptosis, autophagy, senescence, cell growth, and motility (35). The context-specificity of this feedback mechanism appears to be related to disease stage and this warrants further study, especially with regards to apoptosis and autophagy, since subtle changes in the ratio of pAKT to pSMAD can have both pro- or anti-apoptotic effects (39, 40). A proposed model of this pathway is shown in Supplementary Figure 2. In a parallel study we have reported the effects of modification of this pathway using pharmacological antagonism and genomic techniques and found VIC phenotype transition can be controlled by the PI3K/AKT/mTOR pathway (41). This suggests both canonical and non-canonical components of the TGFβ signaling pathway can control VIC phenotype.

The RAS-MEK-ERK pathway was also found to be altered in the datasets. ERK1/2 signaling has been shown to be associated with MMVD in both dogs and humans, either through TGFβ-related or serotonergic signaling (42–47). The data presented here indicates some level of both activation and inhibition of this pathway with the decrease in ERK1-p44/42 MAP kinase phosphorylation, increase in total ERK8, and increased MAP3K1/MEKK1 phosphorylation in some diseased cells. Previously, we have shown that activation or inhibition of serotonin-induced ERK1/2 signaling has no effect on quiescent or diseased cells (16). Phosphorylation of ERK1/2 is known to result in the activation of a variety of transcription factors including CREB and c-fos, as well as cell cycle regulatory transcription factors such as Elk-1 and Sep-1a (48, 49). The pathway can also activate transcription factors that regulate cell survival such as Bim and FasL for inducing apoptosis (50). Inhibition of the MAPK-ERK pathway *in vivo* has been shown to attenuate aortic valve disease progression in Emilin1-deficient mice (51). However, looking at this pathway in the context of TGFβ-induced signaling has not been performed and studies using more specific ERK1/2 inhibitors or investigating phosphorylation changes in this pathway in quiescent cells treated with TGFβ1 would be beneficial.

Changes in phosphorylated Rac1/cdc42 were also identified with a significant increase in diseased cells. Much is known about Rac1/cdc42 in cancers such as breast and pancreatic. Interacting with TGFβ, Rac1 drives endothelial-to-mesenchymal transition (EndoMT) in cancer, in which endothelial cells lose their polarity and cohesiveness and acquire the morphology and migratory properties of fibroblasts (52). To what extent Rac1 might contribute to MMVD is unknown, but EndoMT is recognized to occur and may be an additional source of aVICs (14, 53). Use of inhibitors such as ML141 or a more targeted approach using Rac1 siRNA could be used to further investigate the role of this pathway in MMVD, in particular the effects on expression of key EndoMT transcription factors SNAIL, TWIST, ZEB, and AP-1. On the canonical side of the TGFβ signaling pathway, phosphorylated SMAD2 protein expression was found to be increased, which is also seen, with concurrent increased TGFβ protein expression, in human MMVD (21). SMAD2 expression is thought to be one of the pathways that drive VICs into their activated myofibroblast phenotype. While the downstream events of SMAD2 in MMVD are not fully understood, a pathway has been suggested by Thalji et al. (26) (Supplementary Figure 3).

While we have shown modification of PI3K/AKT/mTOR will affect cell phenotype, with pSMAD2 increased in aVICs, we decided to target it for inhibition using siRNA and the SMAD7 agonist asiaticoside. SMAD7 inhibits SMAD2 phosphorylation and prevents the formation of a complex with SMAD4, stopping SMAD2 from entering the cell nucleus (26). The genes examined as outputs encoded for α-SMA, SM-22, and MHY10, as these markers are commonly expressed at high levels in aVICs (11, 16, 54). We observed the three genes encoding for these myofibroblast markers were significantly decreased after SMAD2 antagonism to an expression level similar to that found in qVICs, suggesting phenotype reversal. Antagonism reduced phosphorylated SMAD2 and αSMA expression while maintaining VIM expression, confirming retention of a mesenchymal phenotype (10). Furthermore, the effects on expression of pERK1/2, pS6K, and pAKT identified levels of cross-talk between the SMAD, MAPK, and PI3K signaling pathways. These data confirm the effectiveness of AST and the SMAD2 siRNA at the dose used in reducing, but not in abolishing, pSMAD2 protein expression, likely preserving normal VIC function. In cardiac models of myocardial disease, SMAD2 inhibition protects against cardiac dysfunction, preventing cardiac fibrosis and cardiomyocyte hypertrophy, raising the possible therapeutic options for treating myocardial and valvular disease by targeting canonical TGFβ signaling (55, 56).

However, the complexity of TGFβ signaling needs to be considered in the development of novel therapeutics. Changes in expression of proteins in the MAPK and PI3K pathways in aVICs shows there is a link between these non-canonical pathways and canonical SMAD signaling, and as we have previously reported, antagonism of PI3K will control disease phenotype (41). The intricacies of this cross talk in disease are best understood in cancer and cancer models, with little information on MMVD. Cross-talk between the canonical SMAD pathway and PI3K pathway has been reported for various cell types including stem cells and cancer cells. This signaling is found to be complex and can either inhibit or stimulate depending on the circumstances. For example, in human embryonic stem cells when PI3K is in abundance, SMAD2 and SMAD3 activate the expression of the pluripotency gene *NANOG* to maintain self-renewal. However, low PI3K activity switches SMAD2/3 signaling to direct cell differentiation (57). Normal mitral valve function itself is dependent on the balance between the MEK/ERK1/2 and SMAD, which is further regulated by Filamin-A (24). Mutations in the Filamin-A gene cause congenital valvular defects and progression to myxomatous mitral valve disease in human subjects and mouse models. Phosphorylated ERK1/2 inactivates the SMAD pathway, preventing build-up of SMAD2/3 in the nucleus, permitting a balance in transcriptional activities (24). This mutation causes a decrease in ERK1/2 activation alongside a marked increase in activation of SMAD2/3. The changes in protein expression for ERK1/2 and SMAD found in the Filamin-A knockout mice do match the changes observed in the canine aVICs in the current study but inhibiting SMAD did not increase expression of ERK1/2. Further work is now needed to examine these relationships in MMVD in more detail.

## 5. Conclusion

Myxomatous mitral valve disease is associated with changes in both the canonical SMAD and several of the non-canonical parts of the TGFβ signaling pathway, particularly PI3K/AKT/mTOR, indicating likely contribution to disease pathogenesis. Antagonizing SMAD2 expression has a beneficial effect on transitioning disease cells back to a normal phenotype, while maintaining their inherent mesenchymal characteristics, and also affects protein expression in the non-canonical components of the TGFβ pathway. Further studies are required to identify additional downstream targets that may then have future novel therapeutic value. In addition, *in vitro* studies using appropriate rodent models would be needed.

## Data availability statement

The original contributions presented in the study are included in the article/Supplementary material, further inquiries can be directed to the corresponding author.

## Ethics statement

The animal studies were approved by VERC R(D)SVS University of Edinburgh. The studies were conducted in accordance with the local legislation and institutional requirements. Written informed consent was obtained from the owners for the participation of their animals in this study.

## Author contributions

BC and VM secured the funding. BC, GM, and VM contributed to the conception and design. GM, AM, and QT carried out the experimental work. BC, GM, and AM wrote the first draft. GM, AM, QT, and VM revised the manuscript. All authors contributed to manuscript revision and editing and approved the submitted version.

## Supplementary material

Supplementary material 1 Full moon Biosystems TGFB Array Protocol.

Supplementary material 2 Protein immunoblotting protocol (western blotting).

Supplementary Figure 1 Example of the Fullmoon TGFβ phospho array profiler indicating individual antibody spots, sets of replicates of the same antibody, and blocks of different antibody.

Supplementary Figure 2 Theorized model of negative feedback loop in MMVD. In early stages of MMVD, there is an increased expression throughout the PI3K pathway leading to the activation of transcription factors involved in apoptosis and autophagy. In the later stages of MMVDS6K, expression is increased and phosphorylates IRS-1, targeting it for degradation. This then prevents IRS-1 from acting on the downstream PI3K pathway, leading to decreased expression throughout the PI3K pathway.

Supplementary Figure 3 Simplified working model of alterations in TGF-β signaling in MMVD affecting canonical SMAD. Red, increased expression and green = reduced expression; BAMBI, BMP and activin membrane-bound inhibitor homolog (*Xenopus laevis*); CREB5, Cycle AMP-responsive element-binding protein 5; DAB2, Disabled homolog 2; FOS, FBJ murine osteosarcoma viral oncogene homolog JUN, Jun proto-oncogene; SIK1, Salt-inducible kinase 1; TGF-β2, transforming growth factor-beta; TGF-βR, transforming growth factor-beta receptor; TGIF1, TGF-β-induced factor homeobox 1 [adapted from (26)].

Supplementary Table 1 OD, 260/280, and amount of sample used for analysis for each of the samples used in the Full Moon TGFβ MAP kinase signaling antibody array.

Supplementary Table 2 Antibodies used for protein immuno-blotting.
