# Supplementary material for: TGF-β phospho antibody array identifies altered SMAD2, PI3K/AKT/SMAD, and RAC signaling contribute to the pathogenesis of myxomatous mitral valve disease
Source: Front Vet Sci. 2023 Oct 16;10:1202001. doi: 10.3389/fvets.2023.1202001 (PMC10613673; doi:10.3389/fvets.2023.1202001)
Supplement: Supplementary material 1 — Full moon Biosystems TGFB Array Protocol. [file Data_Sheet_1.docx]

**Supplementary File 1**

**Full moon Biosystems TGFB Array Protocol**

https://www.fullmoonbio.com/product/tgfb-phospho-antibody-array/

Once VIC confluence was reached for each sample, all samples steps were performed at the same time. Briefly, cells were washed 5 times with ice cold PBS, covered with 1ml of fresh ice cold PBS, scraped off the dish surface, pipetted into a 1.5mL Eppendorf and centrifuged at 5000g for 5 minutes. Pelleted cells were suspended in 100-200µL lysis buffer (with 10µL per mL Invitrogen phosphatase inhibitor added to it) and snap frozen in liquid nitrogen and stored at -80°C for later use (no longer than 1 week). Samples were thawed on ice before having cell lysis beads added, vigorously vortexed for 1 minute and incubated on ice for 10 minutes (repeating this 5 times for each sample) to disrupt and lyse the cells. Samples were centrifuged at 10,000g (table top centrifuge) for 5 minutes at 4°C before all liquid being transferred to a clean Eppendorf, then centrifuged at 18,000g for 20 minutes at 4°C (Eppendorf 5417R). The top clear layer of supernatant was transferred to new labelled Eppendorf tubes. Columns for protein purification were prepared by adding 650µL of labelling buffer to each column and thoroughly mixing and leaving at room temperature for 1 hour before centrifuging at 750g for 2 minutes to remove excess liquid. One hundred µL of each protein was carefully pipetted into the columns before being centrifuged at 750g for two minutes and flow through collected. This purified protein was then quantified and purity checked using the NanoDrop 1000. Nine out of twelve samples were slightly to low in yield for analysis so were concentrated using a speedvac vacuum centrifuge until all samples were suitably concentrated (after 30 minutes of centrifuging). Sample concentrations are shown in Table 3.

Sufficient quantities of each sample were used to provide 60 OD of protein (summarised in Table 2) and pipetted into a new tube, and labelling buffer added to give a total volume of 75µL. Three µL of biotin were added to each sample and incubated at room temperature for 2 hours, with vortexing every 10 minutes. 35µL of stop reagent was added to each sample mixed by vortexing and centrifuged briefly before being incubated at room temperature for 30 minutes with vortexing.

Array slides were blocked with blocking solution with shaking for 45 minutes before being washed in dH_2_O (45ml dH_2_O in a conical tube with a slide and shaken vigorously (repeated 10 times)). Samples were mixed with 6ml of coupling solution and placed onto one slide each and incubated for 2 hours at room temperature with gentle shaking on an orbital shaker. Slides were then washed in wash solution (3 times for 10 minutes each) followed by washing in dH2O as previously. 30ml detection buffer (15µL of 2mg/mL Cy3-streptavidin added to 60mL detection buffer (720mL in total)) was poured onto each slide and incubated at room temperature on an orbital shaker for 20 minutes. Each slide was then again washed in wash solution (3 times for 10 minutes each) followed by washing in dH_2_O. All slides were dried by centrifuging in a conical tube at 1300g for 10 minutes and packaged appropriately before being sent to Full Moon Biosystems for slide scanning.

Slide results were reported back and images of each slide were analysed in ImageJ. A free plugin array profiler was installed and used to analyse blocks of antibodies on the array, with 6 blocks per array slide (Figure 1). Once all slides had the signal intensity of the antibody dots measured and a background for each slide had been measured, R statistical package was used to organise, average signal intensity and subtract background for future analysis.

Median values for all the antibodies for each slide were calculated and used to normalise the data. This normalised data was then used to calculate fold change and statistical analysis between total protein and phospho protein. For total protein the average for normal and then diseased VICs was calculated. The average disease value was then divided by the average normal value to get a fold change difference for the total protein. Two-tailed t-test assuming equal variance was performed comparing normal to diseased median normalised values with p>0.05 regarded as significant. To analyse the phosphorylation for the normalised total protein values the phosphorylated antibody was divided by the corresponding total antibody in the same sample, ensuring that this matched the amino acid sequence that each antibody was targeted against. These phosphorylated values were then averaged for normal and diseased VICs and a t-test was performed comparing the two groups for each of the phosphorylated proteins.
