# Supplementary material for: TGF-β phospho antibody array identifies altered SMAD2, PI3K/AKT/SMAD, and RAC signaling contribute to the pathogenesis of myxomatous mitral valve disease
Source: Front Vet Sci. 2023 Oct 16;10:1202001. doi: 10.3389/fvets.2023.1202001 (PMC10613673; doi:10.3389/fvets.2023.1202001)
Supplement: Supplementary material 2 — Protein immunoblotting protocol (western blotting). [file Data_Sheet_2.docx]

**Supplementary File 2**

**Protein immunoblotting protocol (western blotting)**

Where necessary reactions (e.g. signalling pathway inhibition) were terminated by the addition of 50-µl ice-cold radioimmunoprecipitation assay buffer (50mM Tris (pH 7.4), 150 mM sodium chloride, 2% (v/v) NP 40, 0.25% (w/v) sodium deoxycholate, 1mM EGTA (ethylene glycol-bis(β-aminoethyl ether)-N,N,N',N'-tetraacetic acid), 10mM sodium orthovanadate, 0.5mM phenylmethylsulfonyl fluoride, chymostatin (10 mg/ml), leupeptin (10mg/ml), antipain (10mg/ml) and pepstatin A (10mg/ml)) and kept on ice for 30 min to facilitate the extraction of cellular proteins. The samples were centrifuged at 12,000g for 15min and the resulting supernatants containing the solubilized proteins were used for WB analysis. Protein content of the samples was determined by the Micro BCA Protein Reagent Kit (Pierce, IL, USA). Samples (50µg) were educed and electrophoresed on 10% sodium dodecyl sulfate-polyacrylamide gel electrophoresis resolving gels under reducing conditions. The resolved proteins were transferred to polyvinylidene difluoride (Millipore, Watford, UK) and blocked at room temperature with 10% (w/v) non-fat dried milk in phosphate-buffered saline (PBS)/Tween 20 (v/v 0.1%) under constant agitation. Primary antibody was incubated with the blot for at least 1hr at room temperature. The blots were then washed in PBS/Tween before incubating with goat anti-rabbit IgG HRP in 5% (w/v) non-fat dried milk for 1hr with constant agitation. For each antibody examined activation was determined by reactivity with the antibody to the dually phosphorylated protein and compared to the control antibody (β-Actin or Vinculin) or the total protein expression, and a ratio was calculated. The blots are thoroughly washed and then incubated with enhanced chemiluminescence (ECL) reagent (Amersham Life Sciences, UK) and exposed to film. Image J software is used to attribute densitometry values to quantify the results.
