# Supplementary figures and images for: TGF-β phospho antibody array identifies altered SMAD2, PI3K/AKT/SMAD, and RAC signaling contribute to the pathogenesis of myxomatous mitral valve disease
Source: Front Vet Sci. 2023 Oct 16;10:1202001. doi: 10.3389/fvets.2023.1202001 (PMC10613673; doi:10.3389/fvets.2023.1202001)

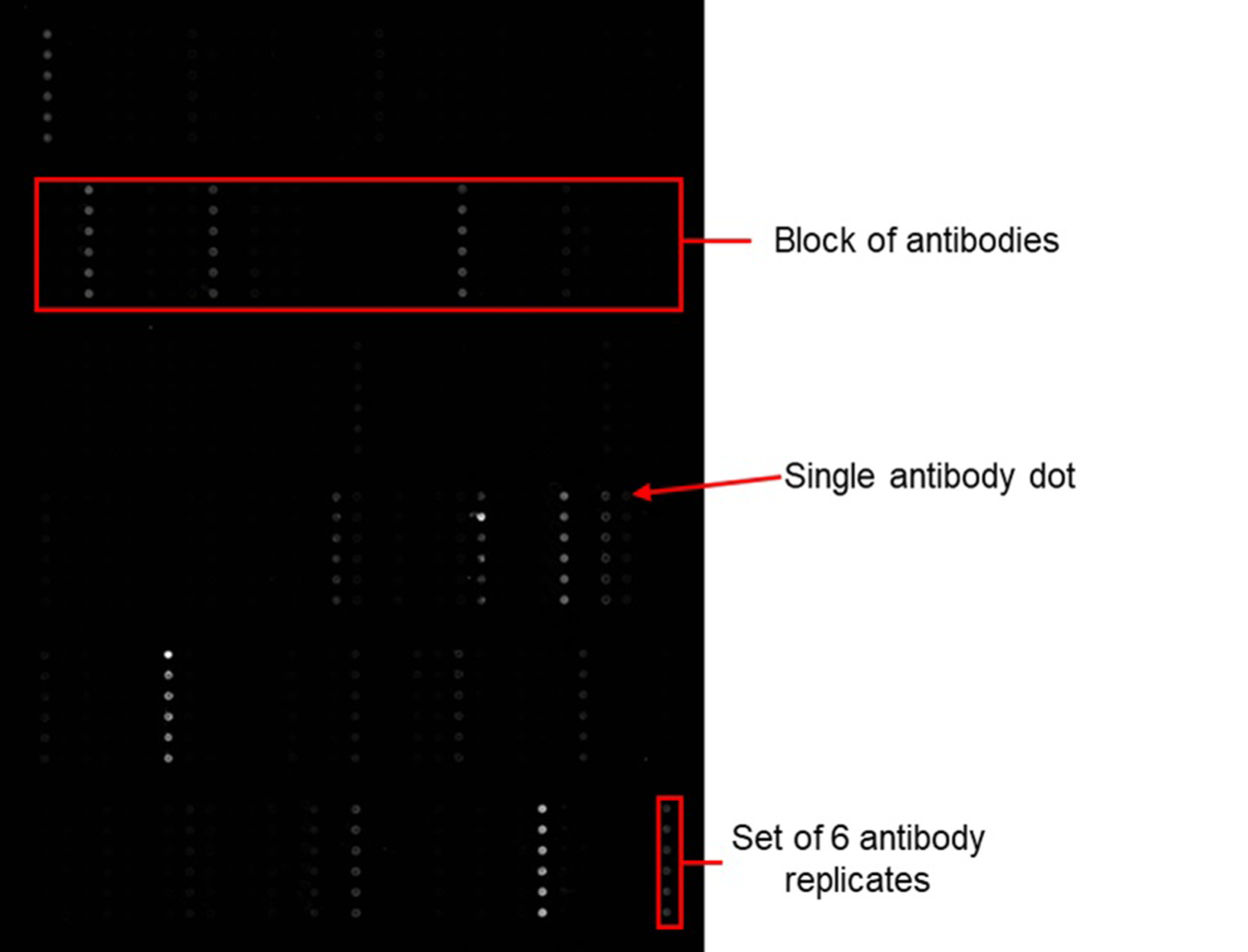

Supplement: Supplementary Figure 1 — Example of the Fullmoon TGFβ phospho array profiler indicating individual antibody spots, sets of replicates of the same antibody, and blocks of different antibody. [file Image_1.TIF]

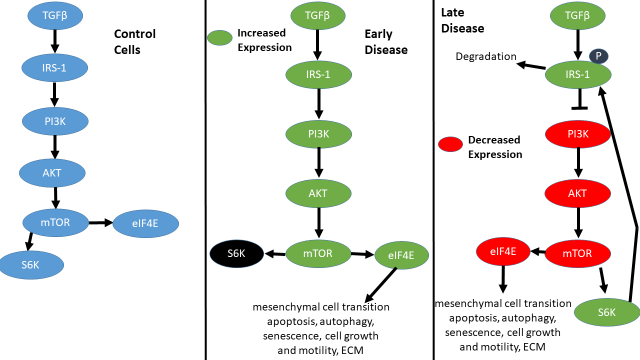

Supplement: Supplementary Figure 2 — Theorized model of negative feedback loop in MMVD. In early stages of MMVD, there is an increased expression throughout the PI3K pathway leading to the activation of transcription factors involved in apoptosis and autophagy. In the later stages of MMVDS6K, expression is increased and phosphorylates IRS-1, targeting it for degradation. This then prevents IRS-1 from acting on the downstream PI3K pathway, leading to decreased expression throughout the PI3K pathway. [file Image_2.TIF]

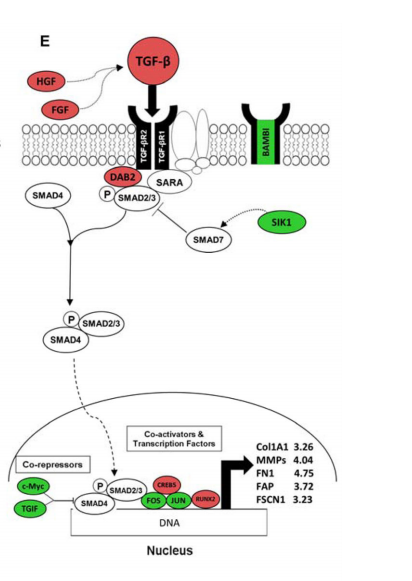

Supplement: Supplementary Figure 3 — Simplified working model of alterations in TGF-β signaling in MMVD affecting canonical SMAD. Red, increased expression and green = reduced expression; BAMBI, BMP and activin membrane-bound inhibitor homolog (Xenopus laevis); CREB5, Cycle AMP-responsive element-binding protein 5; DAB2, Disabled homolog 2; FOS, FBJ murine osteosarcoma viral oncogene homolog JUN, Jun proto-oncogene; SIK1, Salt-inducible kinase 1; TGF-β2, transforming growth factor-beta; TGF-βR, transforming growth factor-beta receptor; TGIF1, TGF-β-induced factor homeobox 1 [adapted from (26)]. [file Image_3.TIF]
