# Supplementary material for: TGF-β phospho antibody array identifies altered SMAD2, PI3K/AKT/SMAD, and RAC signaling contribute to the pathogenesis of myxomatous mitral valve disease
Source: Front Vet Sci. 2023 Oct 16;10:1202001. doi: 10.3389/fvets.2023.1202001 (PMC10613673; doi:10.3389/fvets.2023.1202001)
Supplement: Supplementary Table 1 — OD, 260/280, and amount of sample used for analysis for each of the samples used in the Full Moon TGFβ MAP kinase signaling antibody array. [file Table_1.docx]

**Supplementary Tables**

**Supplementary Table 1. Significantly (P>0.1) altered total protein changes in the TGFβ pathway**

**Supplementary Table 2. Significantly (P>0.1) altered phosphorylation in proteins in the TGFβ pathway high fold change**
